# Self-efficacy in mass casualty incident training using mixed reality

**DOI:** 10.1186/s41077-026-00456-5

**Published:** 2026-07-01

**Authors:** María del Carmen Cardós-Alonso, Miguel Inzunza, Lina Gyllencreutz, Salvador Espinosa-Ramírez, Ana María Cintora-Sanz

**Affiliations:** 1 Emergency Medical Service of the Community of Madrid (SUMMA112), Madrid, Spain; 2 Faculty of Nursing, Physiotherapy and Podiatry, Complutense University of Madrid, Madrid, Spain; 3 Unit of Police Work/Research Unit, Umeå University, Umeå, Sweden; 4 Department of Nursing, Umeå University, Umeå, Sweden; 5 Department of Diagnostics and Intervention, Umeå University, Umeå, Sweden

**Keywords:** Disaster response, Mass casualty incidents, Emergency medical services, Simulation, Self-efficacy, Mixed realities

## Abstract

**Introduction:**

Throughout Europe, Emergency Medical Services (EMS) rely heavily on Medical First Responders (MFR) to ensure early stabilization and appropriate handling of complex Mass Casualty Incidents (MCI). Mixed Reality (MR), also known as Augmented Reality (AR), provides interactive training by integrating real environments with digital components. This technology enables MFR to practise complex decision-making and hands-on interventions in high-stress, simulated scenarios. However, assessing its genuine effectiveness remains challenging, as true competency is often difficult to demonstrate outside real-life emergencies. This study aims to measure pre- and post-test changes in perceived self-efficacy among MFR following a targeted MR intervention focused specifically on the initial management of MCI.

**Method:**

A pre-test/post-test survey design was employed with 274 MFR who underwent detailed MR training simulating complex, early-stage MCI scenarios. To analyze the intervention’s precise impact on participant confidence, data were analyzed using both within- and between-subjects ANOVA, providing information about the change in self-efficacy across the cohort.

**Results:**

The analysis revealed a statistically significant increase in perceived self-efficacy among the 274 participants following the training (F(1,270)=169.36, *p<*.01, η^2^=.385). The mean score increased from 34.45 (SD=6.27) to 38.38 (SD=5.40), representing an absolute increase of 3.93 points. This corresponds to an 11.41% increase relative to baseline scores and an 8.19% increase when calculated against the maximum possible score (48 points), indicating a strong practical effect of the intervention. The MR intervention could detect change and indicate a positive influence on confidence and how a combined approach including MR training can improve MFR perceived preparedness.

**Conclusion:**

Findings suggest that MR training significantly enhances MFRs' perceived self-efficacy in managing MCIs. Consequently, integrating immersive MR technologies into emergency training programs may represent a valuable complementary strategy for strengthening organizational disaster preparedness and improving MFR confidence and decision-making skills in high-stakes situations.

**Supplementary Information:**

The online version contains supplementary material available at https://doi.org/10.1186/s41077-026-00456-5.

## Introduction

Mass casualty incidents (MCI) and disasters, both man-made and natural, are unexpected events that occur around the world, causing alarming impact, terror, and suffering. As emergency medical services (EMS) have been developed and professionalized, there has been a shift from improvisation to anticipation and specific training, which seeks to minimize the impact on the community and reduce the morbidity and mortality of victims [1], measuring self-efficacy is not a direct indicator of mass casualty victim survival. However, it does measure a different and complementary impact related to the behaviour and practical competencies of trained personnel.

Preparing emergency teams to deal with MCI is a considerable challenge, as recreating such a scenario can be complicated. The challenges of disaster management are related to the ability to manage uncertainty and unforeseen situations, and the ability to use structured processes to respond flexibly. The ability to adapt mindsets, operational procedures, decision-making processes and command structures from both an organisational and individual perspective is of crucial importance. Medical, non-medical and internal factors, such as staff and managerial stress, influence health emergency leadership and decision-making [2, 3]. Considering the significant financial challenges associated with training large numbers of participants in disaster exercises, it is imperative to explore effectively training methods for EMS professionals [4] that can achieve results equal to or even superior to traditional learning models. This is where virtual reality (VR) technology could play a key role in the context of healthcare simulation education [5, 6]. It is important to understand the specific advantages that VR and MR technologies can offer in training sessions. In VR, users are fully immersed in a computer-generated environment, where the physical world is entirely hidden. This creates a controlled setting where users can safely explore and interact with the virtual elements that imitate real-world objects, though without physical object interaction [7, 8]. Virtual reality simulation not only promotes the development of technical skills, but also fosters cross-cutting competencies such as confidence, leadership, and collaborative work. This is because it provides a safe space where students can practise interventions and correct mistakes without putting patients at risk [9]. MR settings occur in the physical environment but include virtual objects that engage with the user and the real world while maintaining the physical environment. MR integrates virtual and physical elements into a shared environment where digital content and real-world objects coexist and interact. For example, in a simulated accident involving multiple casualties, users may view a virtual emergency scene through a head-mounted display while hearing environmental sounds or clinical instructions through headphones. On the other hand, AR scenes occur in the real world and include non-dominant virtual objects that interact with the user and/or the real environment without entirely obscuring it.

At the same time, they can physically interact with a real manikin equipped with sensors, allowing their actions to influence the unfolding virtual scenario. This combination enables learners to engage multiple senses—such as sight, touch, and hearing—within a cohesive experience that blends and synchronizes virtual and real-world components [10].

The 18-partner European MED1stMR project has developed an innovative MR training solution for first medical responders, such as nurses, paramedics and doctors. The solution is intended to improve their preparedness for incidents involving multiple victims. Following two years of prototyping and development, the project team has created an cutting-edge platform solution ready to be evaluated by users [11, 12].

MED1stMR training is based on virtual simulations that allow training in high-risk situations, such as MCIs, enhancing the performance of those involved. The system combines a training scenario framework, MR technology with VR-enabled manikins

and wearable devices for measuring biosignals, as well as intelligent scenario control that monitors participants' stress levels in real time using a control panel for the trainer and automated systems based on artificial intelligence.

**Fig. 1:**
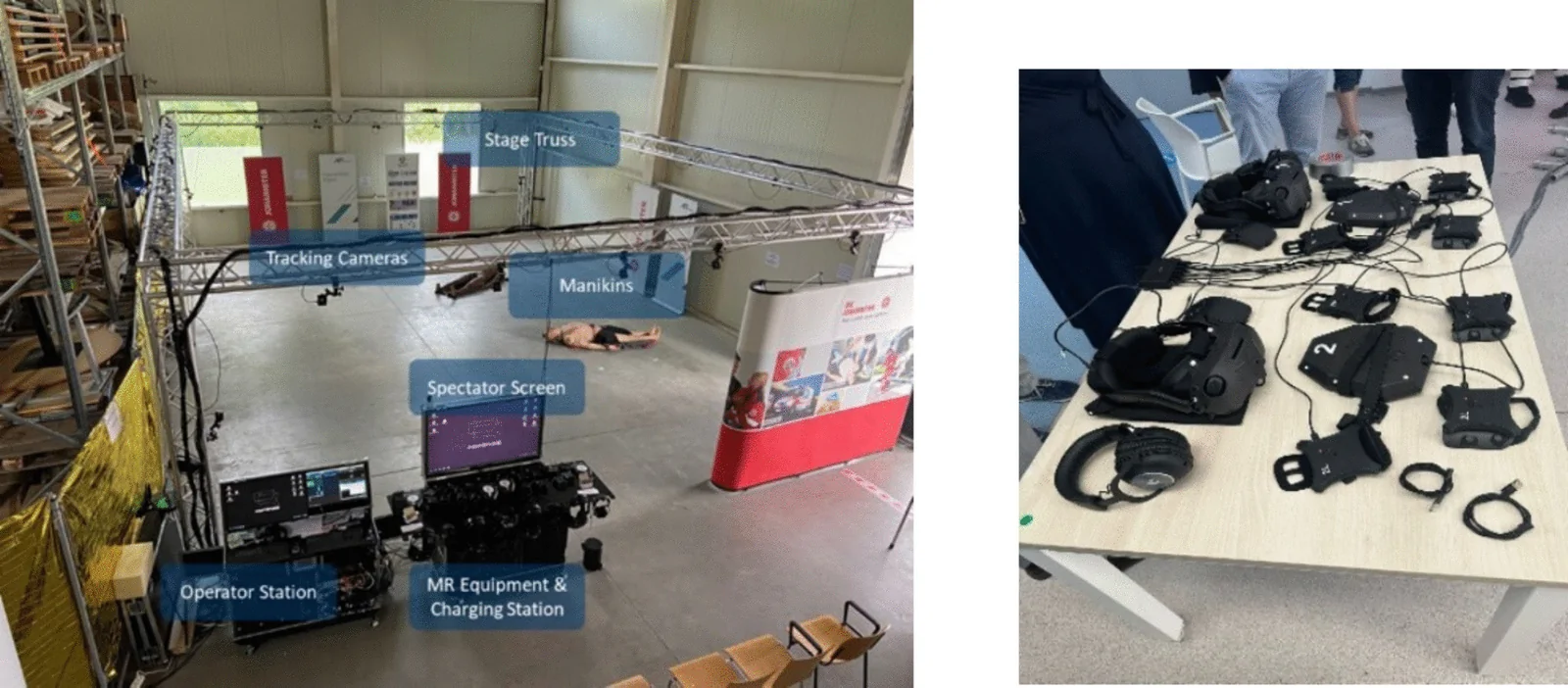
The left-hand photo illustrates the core components and overall setup of the mixed reality training system. The photo on the right presents the sensors employed in the system

**Fig. 2:**
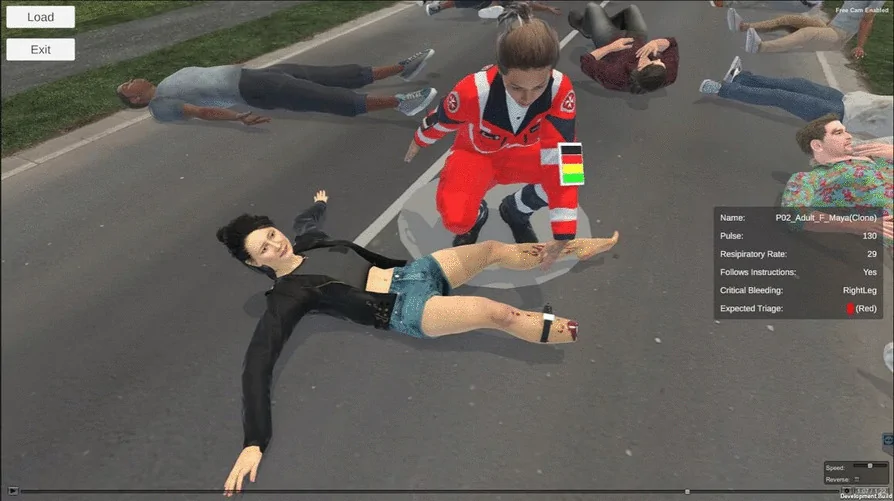
Patient Information highlighted

**Fig. 3:**
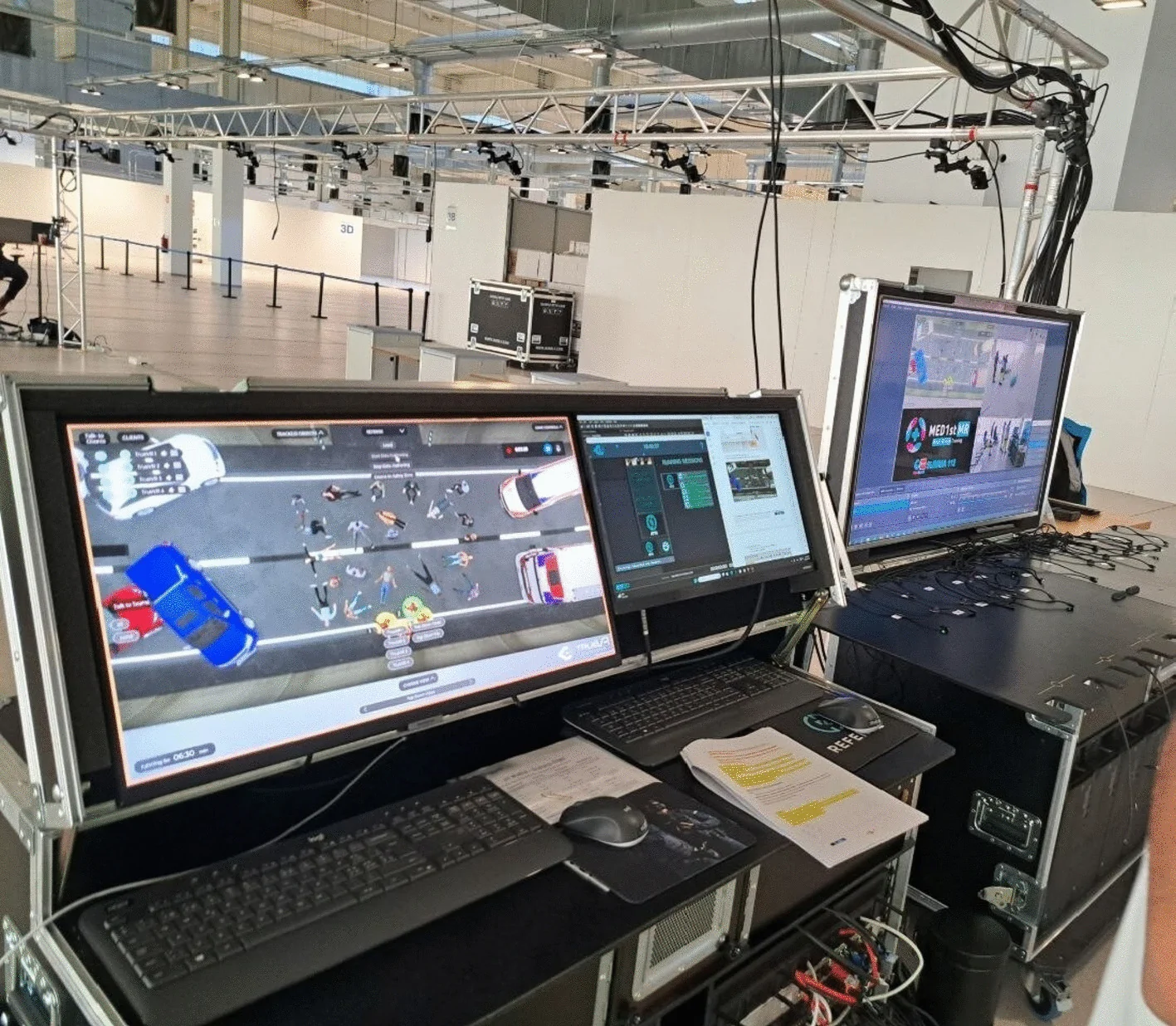
Dashboard control during trainings

Between July 2023 and January 2024, six field tests were conducted in six countries, with a total of 274 participants. Each session allowed four people to train simultaneously in the MR environment, facing two scenarios: one on the road and one in a tunnel, both simulating a bus accident with 20 casualties. The aim was to improve the organizational, communication, and triage skills of the participants, who interacted via headphones and assessed the casualties, represented as virtual avatars or physical manikins, allowing for direct assessment of breathing, pulse, level of consciousness, and critical bleeding (supplementary files and Fig. 1, 2 and 3).

A systematic review of the preparation and training of medical first responders for MCI [13] highlights the potential of VR/MR, although these technologies are not yet widely used in MCI training. Similar outcomes were found in another scoping review [14]. The research further notes that less than a third of the included studies utilised observation of the performance as an evaluation method. Numerous studies relied on knowledge tests or self-assessments, such as confidence, despite their limited predictive value for actual performance [15–17].

This research project aims to evaluate the impact of a training intervention using mixed reality (developed by the MED1stMR project) on the self-efficacy of emergency medical services professionals and medical students in the initial management of incidents involving multiple victims.

## Methods

### Design

The study used a pre- and post-test, adapting a validated survey known as the “SELF-EFFICACY scale for medical first responders (MFR) in MCI” (SESMCI); we modified it to use only the first 8 items [18, 19] measuring how self-efficacy changed following a training intervention with MR technology in initial MCI management. The study was conducted as part of a multinational European project involving participants from several countries.

### Instrument and development

The SESMCI instrument originally consisted of 13 items covering all aspects of an MFR professional’s MCI management (ANNEX I in supplementary files) [18, 19]. Responses were provided on a six-point scale, ranging from 1 to 6. For this study, only the first eight items of the scale were selected (8 out of 13 questions), as they corresponded with the training's learning objectives, particularly those related to practising initial first triage skills. Consequently, it was necessary to examine the psychometric properties of these eight items to confirm their validity and suitability within the context of this study (see Data analysis section).

Given the multinational study context, the SESMCI instrument was developed and validated in Spanish. The items were first translated into the language of the participants' country and then retranslated into the original language by bilingual experts to ensure semantic and conceptual equivalence. An adaptation process was then carried out to ensure cultural and contextual relevance, which included review and minor modifications beyond direct translation (with the help of researchers, experts, and professionals in the field of disaster medicine) [20, 21].

### Training

Within the MCI training, 4 participants are involved. We proceed to explain the intervention, in which they are grouped in pairs. In each pair, one carries the communications device to report the injured to the communications centre, and to request resources; the other is in charge of classifying the victims with triage cards and performing life-saving manoeuvres. As there are 20 victims, they separate and each classifies 10 victims and reports.

### Data collection

To ensure anonymity and enable data linkage across time points, each participant was first assigned a unique random Identification number, that they had to use each time they completed different surveys. Data were then collected at two stages using the same procedure. Before MR training, participants received an online survey containing background information and the first eight items from the SESMCI instrument. Prior to completing the online survey, they were informed about the study’s purpose, their right to withdraw at any time, and the voluntary nature of participation. After completing the initial survey (T1), participants engaged in two MR training scenarios designed to simulate early-stage mass casualty incidents. Immediately following the training, they completed the same online survey a second time (T2). This procedure ensured consistency in data collection and allowed for accurate measurement of changes in perceived self-efficacy.

### Data analysis

The instrument was shortened and therefore required analyses to investigate if its suitability in terms of validity and reliability remained intact. Confirmative factor analysis (CFA) was used to develop models based on the 8 items for both T1 and T2. The fit of the models was investigated with several fit indices. The following fit indices and thresholds were used to provide guidance when evaluating the models: the Chi-square test being not statistically significant (p >.05) although it is considered an overly strict test [22]. The comparative fit index (CFI) with a minimum of.90 indicated an acceptable fit of a model. A root mean square error of approximation (RMSEA) of.08 or lower indicated an adequately fitting model. A standardized residual mean square (SRMR) of 0.08 or less demonstrated an acceptable fit for the model [23–25].

These analyses were performed with Mplus version 8 [26]. The reliability of the 8-item measure was evaluated with coefficient omega [27]. A composite score was developed from the self-efficacy measure based on eight items.

To investigate change before and after scores in self-efficacy, ANOVA within-subjects and between-subjects analyses were performed with selected variables providing background information from the participants [28]. Variables included in these analyses were gender, age, country (language), occupation and professional experience. SPSS version 29.0. was used to conduct several of these analyses [29]. Because some background variables contained missing data, the number of participants varies slightly across analyses. We report both individual items and composite scores: individual items usually include the full sample, whereas composite scores require complete data across all items. Participants with missing responses on any composite‑score item were therefore excluded from those analyses.

### Ethical considerations

The study obtained ethical approval through the Med1stMR project from the Ethics Committee of Cultural and Behavioral Studies of Heidelberg University (AZ Beu 2023 1/1).

Study participation was entirely voluntary, and all participants were fully informed of research aims and procedures. They were informed that the purpose of the study was to assess the self-efficacy scale and not to evaluate their performance during the training activities.

After signing an informed consent form, each participant was assigned a randomly generated reference number, which was known only to them. This was done to preserve anonymity when analysing the data.

## Results

The results are presented in three sections: first, the characteristics of the participants; second, the development of the models; and finally, the outcomes of the participants' self-reported self-efficacy before and after the MR training**.**

### Background characteristics

The study recruited 274 participants (paramedics, nurses, doctors, volunteers, and medical students) by convenience sample method from the six field trials conducted within the Med1stMR project (Table 1, ANNEX II: *day/week schedule during the Field Trials)* The cohort represented a heterogeneous group with respect to gender, experience, profession, and volunteerism.

**Table 1:** Demographic and professional characteristics of field trial participants

		N	%
AGE	Age <= 35 years	99	37,4
	Age 36–50 years	113	42,6
	Age > 50 years	53	20,0
GENDER	Female	89	33,7
	Male	175	66,3
JOB EXPERIENCE	< =5 years	80	30,2
	>5 <=20 years	132	49,8
	> 20 years	53	20,0
OCCUPATION	Emergency doctor	31	11,7
	Emergency nurse	45	17,0
	Technician (2 years of education)	52	19,6
	Driver or assistant (less than 1 year education)	56	21,1
	Other doctor	7	2,6
	Medical student	7	2,6
	Other (volunteers, firefighters, police)	33	12,5
	Paramedics (3–4 years of higher education)	34	12,8
Country of participant	Austria	34	12,8
	Belgium	51	19,2
	German	44	16,6
	Greek	44	16,6
	Spain	57	21,5
	Sweden	35	13,2

Recruitment followed a convenience sampling approach, whereby the organizations hosting the field trials in each of the six participating countries were responsible for recruiting participants from within their own organization, according to local procedures and operational prerequisites. As a result, the participant cohort represented a heterogeneous sample in terms of profession, level of experience, gender, and employment status (professional or volunteer), reflecting the diversity of personnel involved in initial MCI management across Europe.

Each organization adapted the practical implementation of the training to fit its local organizational structure and operational context. However, to ensure methodological consistency, all field trials followed the same standardized training protocol, scenarios, learning objectives, and assessment procedures. Adherence to the common study protocol was closely monitored by the project consortium to ensure that the training intervention and data collection were conducted in a comparable manner across all participating organizations and countries. The final sample size was sufficient to evaluate changes in self-efficacy and assess the measurement properties of the instrument.

### Model

The CFA model of the Self- Efficacy scale (SESMCI) based on 8 items at T1 showed an acceptable fit to the data based on several fit indices: significant chi-square (20) =46.44, *p <*.01; Comparative Fit Index (CFI) =.97; Root Mean Square Error of Approximation (RMSEA) =.07; Standardized Root Mean Square Residual (SRMR) =.03.

At T2 again most fit indices of the CFA model of SE based on 8 items showed an acceptable fit to the data, significant chi-square (20) = 47.91, *p <*.01; Comparative Fit Index (CFI) =.95; Root Mean Square Error of Approximation (RMSEA) =.07; Standardized Root Mean Square Residual (SRMR) =.04. Loadings were all significant ranging from.63 -.86 at T1 and.53 -.86 at T2. This indicates supporting evidence of construct validity for the 1-dimensional model of self-efficacy. Reliability calculated as Omega was.92 at T1 and.89 at T2. Composite scores for T1 and T2 were developed from the 8 items and ranged T1 from 13–48 where 48 is the maximum possible score and at T2 from 22–48 where 48 is again the maximum possible score and a higher score indicated higher level of self-efficacy.

Our findings indicate that there was a significant difference in self‑efficacy pre–post training, based on the composite score calculated for participants with complete data across all included items (*n =* 271). A one-way within subjects ANOVA test showed, F (1,270) = 169.364, *p <*.01, η2 =.385. The following observed means were noted at the different timepoints: T1 (M = 34.45, SD = 6.27) and T2 (M = 38.38, SD = 5.40). There is an overall increase of self-efficacy from T1 to T2. Partial eta squared for the whole group is 0.385 (strong effect size) indicating that participants reported higher on the Self-Efficacy Scale in MCI after the MR training intervention. This represents a mean increase of 11.41% from the T1 mean or 8.19 % when calculated relative to the maximum possible score of 48.

### Self-efficacy

**Fig. 4:**
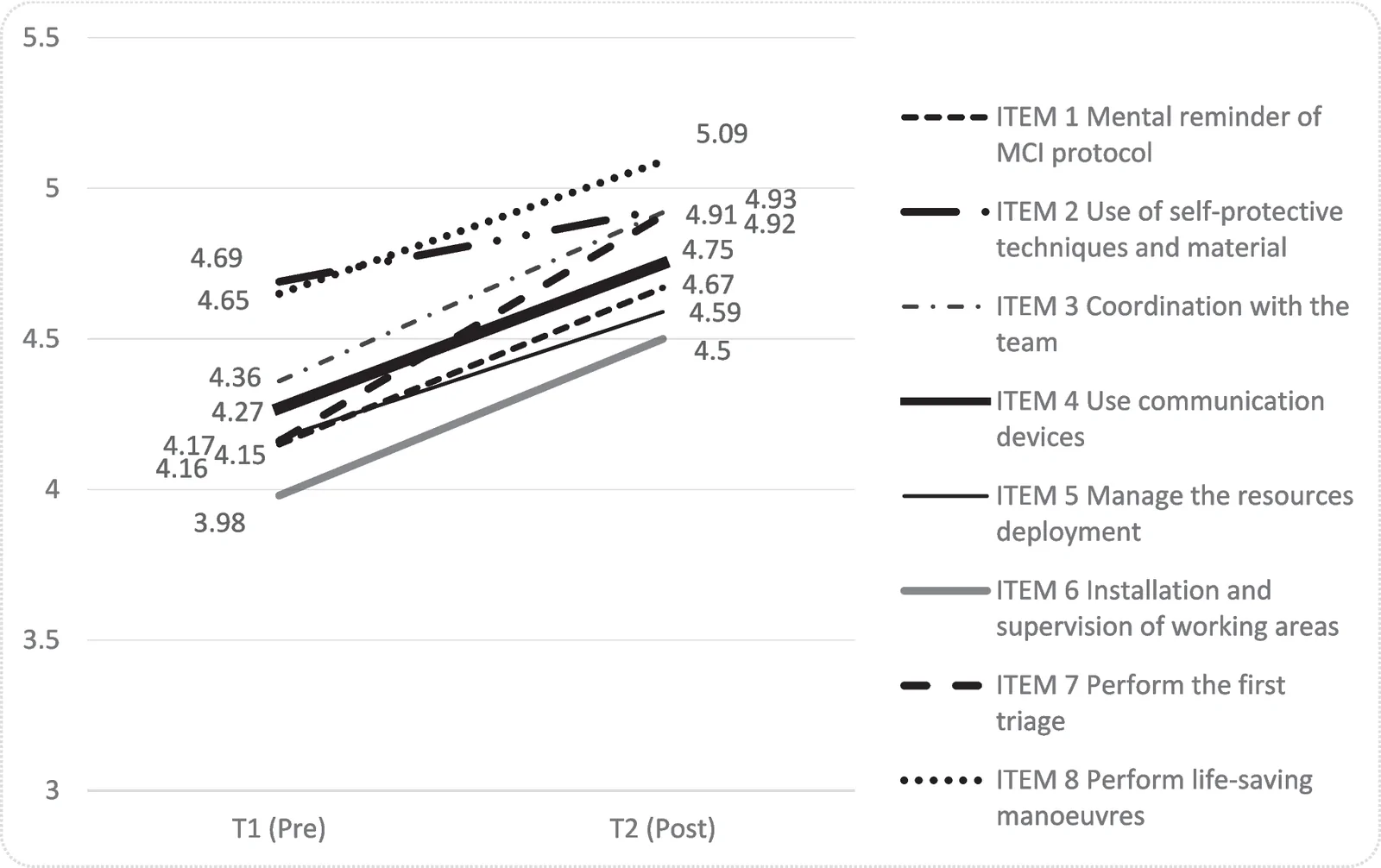
Differences for each item, based on a response format ranging from 1 to 6

The differences in self-efficacy at item level show significant differences (Fig. 4).

The results from the ANOVA indicate increases in self-efficacy in several categories, see Table 2.

**Table 2:** SESMCI scale items showing an increase in mean self-efficacy (>0.70), classified by category and magnitude of increase

Item	Category	Significant increase (>0.70)
ITEM 1: Mental reminder of MCI protocol	Profession	Other physician, not emergency one (0.71)
ITEM 2: Use of self-protective techniques and material		
ITEM 3: Coordination with the team	Gender	Woman (0.78)
	Hours of MCI training	Not received any previous training or course (0.79)
	Profession	Emergency physicians (0.81), other physicians (0.86), and medical students (1.14)
ITEM 4: Use communication devices	Country of participant	Spanish trainees (0.77)
	Profession	Nurses (0.73) and medical students (0.86)
ITEM 5: Manage the resources deployment	Gender	Woman (0.72)
ITEM 6: Installation and supervision of working areas (zoning)	Profession	Medical students (1.00) and paramedics (0.71)
ITEM 7: Perform the first triage	Age	Aged <= 35 (0.85)
	Gender	Woman (0.93)
	Years of work experience	<=5 years (0.81) and between >5 and <=20 years (0.73)
	Hours of MCI training	Not received any previous training or course (1.00) and 1–10 hours of training (0.91)
	Hours of MCI experience	Have not worked in any MCI (0.86) and have worked 1–10 hours in MCI (0.71)
	Country of participant	Austria (0.74), Belgium (0.86), Germany (0.89) and Spain (0.84)
	Profession	Emergency physicians (0.74), technicians (0.75), other physicians (1.00), medical students (1.43), the other category, which includes volunteers, firefighters, police officers, etc., (0.76) and paramedics (0.74)
ITEM 8: Perform life-saving manoeuvres	Profession	Medical students (1.00)

At the item level they were also conducted Independent-Samples Kruskal-Wallis tests. Several differences emerged, for example, for item 5 [Perform and/or supervise the deployment of resources effectively and efficiently], participants from Spain showed a difference of 0.67, a comparative high value relative to the other groups, see Table S3 (Supplementary Material).

Furthermore, using this same test, we found that items 3 (*p=*0.005), 7 (*p=*0.038), and 8 (*p=*0.014) show statistically significant differences with respect to hours of experience in MCI.

### Composite score and group differences

When incorporating group variables, a significant interaction effect could be noted with gender as shown in Figure S5 (Supplementary Material). A within and between-subjects design ANOVA test showed, F (1,260) = 179.21, *p =*.01, η2 =.41. The partial eta squared for the interaction was significant. Females exhibited a significantly higher increase in Self-Efficacy from T1 (32.17) to T2 (37.44) in comparison to males from T1 (35.90) to T2 (39.01) with a value of.04 suggesting a small interaction effect.

When incorporating country of participant, most participants regardless of country exhibited a statistically significant difference in Self-Efficacy from T1 to T2. A within and between subjects design ANOVA test showed, F (1,250) = 132.80, *p =*.01, η2 =.35. There was no significant interaction observed as shown in Figure S6 (Supplemental Material)*.* The significant interaction of gender was still present *p =*.01, η2 =.03, (weaker).

When incorporating occupation most participants exhibited a similar statistically significant difference in Self-Efficacy from T1 to T2, with more increase among medical students. Within and between subjects, the design ANOVA test showed that F (1,216) = 73.21, *p =*.01, and η2 =.25. No significant interaction effect could be observed with any group variable as shown in Figure S7 (Supplemental Material). The significant interaction of gender was not present.

## Discussion

The results show that the original validated instrument developed to measure self-efficacy in traditional non-technological training works well when shortened and used in MR training.

Measuring the impact of training on the survival of MCI victims, which would be a level 4 in Kirkpatrick's model [30] is challenging as such incidents are fortunately rare, making it difficult or nearly impossible to obtain a significant and comparable sample of similar cases.

Self-efficacy is a dynamic, context-specific construct referring to individuals’ perceived capability to perform tasks effectively. In technology-based learning environments, higher self-efficacy is associated with improved problem-solving, persistence, collaboration, and skill application, whereas lower levels are linked to anxiety, impaired decision-making, and reduced resilience [31, 32].

Rooted in Bandura’s self-efficacy theory, this construct highlights the close relationship between perceived competence, motivation, and performance in specific contexts [33]. Although self-efficacy is a subjective measure, it serves as a meaningful predictor of future behaviour and adaptive responses to challenges [32].

In disaster management, self-efficacy is particularly critical for Medical First Responders, especially regarding decision-making, prioritisation, stress management, and communication during mass casualty incidents (MCI) [34]. Prior research indicates that VR training can significantly enhance self-efficacy, triage performance, and response speed [35]. Likewise, MR training offers realistic MCI simulations that enable responders to practise essential prehospital skills under conditions resembling real emergency stressors. Consequently, evaluating the effectiveness of MR training in enhancing Medical First Responders’ perceived self-efficacy remains necessary.

As a result, the focus shifts to assessing the impact of training on the participants themselves. This study provides empirical support for the reliability and conceptual validity of the SESMCI as a measure of self‑efficacy; however, full validation requires comparison with an established gold‑standard measure. Self-efficacy, as defined by Bandura [36], is one's belief in their ability to organise and execute required actions and handle arising situations. Previous research [37, 38] has shown that self-efficacy can be improved through VR training. This study extends that understanding by demonstrating that our validated instrument effectively measures self-efficacy in MR training as well.

Our results demonstrate that self-efficacy significantly improves with MR training, particularly in the context of first triage. Specifically, MR training enhances self-efficacy in key areas such as treatment prioritization, resource allocation, identification of high-risk patients, and the overall confidence in one's ability to act as an effective first responder. Additionally, the MR training helped participants improve their perceived abilities to implement intervention procedures, practise self-protection, coordinate effectively, and use communication devices proficiently.

Similar improvements in performance have been observed in other studies using VR technology. In one study that used VR to train triage in MCI (named GoSaveThem), students reported that they found the training very useful, saying that it improved their ability to perform the task quickly and correctly. They emphasised the importance of incorporating stressors and sensory stimuli, such as smell, hearing and sight [39]. Another study that compared triage training in a real setting with virtual reality found similar performance results, but emphasised the value of using the two methods together [40].

BioSimMER [39] offered immersive emergency simulations utilizing basic motion gestures for navigation, while the Performance Edge platform [41], a novel virtual reality method, allows biofeedback-enabled stress management skills through a training platform that facilitates the practise of cognitive strategies in a diverse, private and immersive training environment. Other research [41, 42] found that participants who engaged in immersive VR training, which utilised motion sensors but lacked haptic feedback and physical simulators, also demonstrated significant performance gains. Our study aligns with these findings but distinguishes itself through the use of a comprehensive MR system that integrates fully immersive VR with haptic sensors for hands, feet, and back, along with headphones, and crucially, a manikin that allows participants to practise life-saving techniques. Unlike other computer-based training systems, our MR training offers a more holistic and tactile learning experience.

Moreover, the TOUCH project introduced an immersive VR application where students wore a head-mounted display (HMD) and navigated a VR environment using a joystick, primarily focusing on diagnosing and treating a traumatic brain injury patient. Notably, researchers from the TOUCH project later found that fully immersed learners made significantly greater gains in knowledge compared to those who interacted with the simulation using a screen-based simulation [43]. These findings further underscore the value of immersive environments in enhancing training outcomes, with our MR system offering an advanced, integrated approach that builds on these earlier technologies.

Although the gold standard for disaster training remains full-scale exercises, several studies suggest that technology-based training methods are effective. This type of training could be considered a complementary tool for comparing two groups, as it could save time and costs [42, 43].

This may also be true for practical skills training, which was always delivered alongside other methods and generally resulted in comparable or lower training effectiveness than approaches involving technology-based methods [42]. Also, the reduced costs of training using technologies such as virtual reality is generally lower than training with full-scale simulations. In the former, only the cost of development and staff time is computed, whereas in live simulation, staff time, set-up, actors, direction, vehicles, etc. are included, which is approximately 13 times more expensive. Depending on the size (number of patients) and scope (extent of departments involved) of the MCI exercise in a hospital, a full-size MCI drill may entail costs between 10,000 and 100,000€. [43, 44].

As Table 2 shows, the perception of self-efficacy improved, as shown in the self-reported data, in all eight items of the SESMCI scale studied (ANNEX I), so that these people, after training, see complex problems as tasks that they have the opportunity to master; they develop a great interest in the activities in which they participate; they develop a solid commitment to their activities and interests; they recover quickly from disappointments and setbacks. In other words, it contributes to improvements in non-technical skills such as situational awareness and avoidance of fixation errors.

Our results showed that men self-reported higher self-efficacy values (Figure S5), which is consistent with other authors [45–47] who have also found that being male could be a predictor of self-efficacy and found statistically differences in self-efficacy as a function of gender. Our data shows that women self-reported a higher growth in self-efficacy than men: 3 points compared to 2 points (Table S3). It has been suggested that this is not due to ability; in fact, there are no significant gender differences in ability measures. This leads us to believe that the differences in self-efficacy are due to women's belief that they cannot achieve certain things [48] (Figure S5).

If we incorporate the country of participants (Figure S6), most of the participants showed a similar difference in Self-Efficacy from T1 to T2. We believe that all Field Trials participants are Medical First Responders who have been trained in this type of MCI. The experience with Mixed Reality has allowed us to increase Self-Efficacy among all countries, indicating that we are well on our way to the universality of MCI performance among European countries, as they all show a similar trend.

When incorporating the trainer's occupation (Figure S7), most participants showed a similar difference in Self-Efficacy from T1 to T2, with a more significant increase among medical students. This shows that any MFR can integrate and use this type of training and that training together to perform together in an MCI is advisable.

### Limitations and future lines of research

This study has several limitations that should be considered when interpreting the findings. First, the pre–post design without a control group limits causal inference, as observed changes in self-efficacy cannot be attributed exclusively to the mixed reality (MR) intervention. In addition, the absence of randomisation introduces the possibility of confounding factors, including prior experience, group dynamics, and contextual differences across field trials.

Second, the outcome measure was based on self-reported self-efficacy rather than objective assessments of clinical or operational performance. While self-efficacy is a relevant and theoretically grounded construct in emergency training, it does not necessarily translate directly into improved performance in real mass casualty incidents (MCI). Furthermore, short-term post-intervention measurements do not allow conclusions regarding the durability or retention of the observed effects.

Third, the SESMCI scale was used in a shortened form (8 of 13 items), which required additional psychometric evaluation. Although acceptable model fit and reliability were demonstrated, the modification may affect comparability with previous validations and limits full confirmation of construct equivalence across contexts.

Fourth, the study relied on convenience sampling across six European countries. Although this resulted in a heterogeneous participant sample, it may limit generalisability to the broader population of medical first responders. Differences in recruitment procedures and local implementation may also have introduced variability not fully captured in the analysis.

Finally, the novelty of MR technology may have influenced participant responses, potentially inflating self-reported gains in self-efficacy (Hawthorne or novelty effects). Future studies should include control groups, long-term follow-up measurements, and objective performance-based outcomes to better evaluate the effectiveness of MR-based training.

Future research should also explore comparative effectiveness across training modalities (MR, VR, AR, and traditional simulation), investigate long-term retention of skills and self-efficacy, and assess how individual and organisational factors influence learning outcomes in immersive disaster training environments.

## Conclusion

Previous research highlighted that general self-efficacy affects the way people interpret training messages. However, how immersive MR technology influences self-efficacy remained unknown. This study provides preliminary evidence that MR training is associated with increased perceived self‑efficacy among medical first responders managing Mass Casualty Incidents (MCI), although causality cannot be definitively established due to the study design. Participants reported improvements in their confidence to perform essential tasks such as triage, coordination, and communication, confirming that MR environments effectively strengthen disaster preparedness. The consistent increase in self-efficacy across professional groups and participating countries suggests that MR training may be adaptable across different European emergency medical service settings. Overall, MR-based training represents a valuable complement to traditional disaster education methods. It offers a safe, scalable, and cost-efficient approach to improving the readiness and confidence of first responders. Future research should continue exploring how immersive training technologies contribute to the development and long-term retention of operational competence in real emergency situations.

## Supplementary Information

Supplementary Material 1.

## Data Availability

Raw data from this study can be performed available at the reasonable request of the authors. https://portal.ait.ac.at/sites/MED1stMR

## Abbreviations

AR: Augmented Reality
EMS: Emergency Medical Services
FTs: Field Trials
MCI: Mass Casualty Incidents
MED1stMR: medical first responders and mixed reality project
MFR: Medical First Responders
MR: Mixed Reality
SESMCI: Self-Efficacy Scale for first responders in Mass Casualty Incidents
VR: Virtual Reality
XR: Extended Reality
