# Supplementary material for: Self-efficacy in mass casualty incident training using mixed reality
Source: Adv Simul (Lond). 2026 Jul 1;11:64. doi: 10.1186/s41077-026-00456-5 (PMC13595628; doi:10.1186/s41077-026-00456-5)
Supplement: Supplementary file 1 [file 41077_2026_456_MOESM1_ESM.docx]

Supplementary material

*Figure S4: “Country Road” Mass Casualty Simulation Scenario*

*Figure S5: Estimated marginal means of self-efficacy in performing mass casualty incidents across time points, by gender.*

*Figure S6: Estimated marginal means of self-efficacy in performing mass casualty incidents across time points, by country of participants.*

*FigureS 7: Estimated marginal means of self-efficacy in performing mass casualty incidents across time points, by occupation.*

*Table S3: Item differences between categories with Independent-Samples Kruskal-Wallis Test.*

| ITEM | CATEGORY | SIGNIFICANT DIFFERENCE (Sig.) |
| --- | --- | --- |
| ITEM 1: Mental reminder of MCI protocol | Hours of MCI training | * |
| ITEM 2: Use of self-protective techniques and material |  | ns |
| ITEM 3: Coordination with the team | Gender  Hours of MCI experience | **  ** |
| ITEM 4: Use communication devices | Gender  Hours of MCI training | *  * |
| ITEM 5: Manage the resources deployment | Gender  Country of participant | **  * |
| ITEM 6: Installation and supervision of working areas (zoning) | Gender | * |
| ITEM 7: Perform the first triage | Gender  Hours of MCI experience | *  * |
| ITEM 8: Perform life-saving manoeuvres | Gender  Hours of MCI experience | *  * |

Notes: ns=not significant at p<.05, *p< .05, **p<.01

ANNEX I

**Self-efficacy in performing mass casualty incidents (SESMCI).**

Below are procedures typically performed in a mass casualty incident (MCI). Please indicate your confidence to achieve them successfully in a real MCI situation.

1: No confidence at all

2: Slightly confidence

3: Some confidence

4: Fairly confident

5: Highly confident

6: Completely confident

| **My degree of confidence in performing the following actions is...** | | |
| --- | --- | --- |
| **Ítems** | | **Degree of confidence** |
| 1. | Mental reminder to fulfil the MCI intervention protocol as I approach the scene. | 1 - 2 - 3 - 4 - 5 - 6 |
| 2. | Proper use of self-protective techniques and/or material at the scene (in order to prevent intrinsic and extrinsic hazards). | 1 - 2 - 3 - 4 - 5 - 6 |
| 3. | Coordination with the team members to initiate appropriate actions, according to my role in the MCI. (distribution of tasks). | 1 - 2 - 3 - 4 - 5 - 6 |
| 4. | Use communication devices correctly according to MCI procedures in each country | 1 - 2 - 3 - 4 - 5 - 6 |
| 5. | Manage the deployment of resources effective and efficiently. | 1 - 2 - 3 - 4 - 5 - 6 |
| 6. | Installation and supervision of working areas (zoning) and deployment of possible structures. | 1 - 2 - 3 - 4 - 5 - 6 |
| 7. | Perform the first triage (classification) within the allocated time and MCI procedures. | 1 - 2 - 3 - 4 - 5 - 6 |
| 8. | Perform life-saving manoeuvres when needed (according to MCI procedures). | 1 - 2 - 3 - 4 - 5 - 6 |

ANNEX II *Day/week schedule during the Field Trials*

| **Phase** | **What** | **Detail** | **Where** | **By whom** | **Minutes** |
| --- | --- | --- | --- | --- | --- |
| **Early Bird Coffee** | Participants arrival | Welcome (coffee and tea)  Signing participants list & consent form(s)  Changing clothes to Operational dress | General meeting room | ICP* and/or Trainer |  |
| **Pre –training**          **45 min** | Welcome & Information | PowerPoint: “Welcome & information” | Research room | ICP and/or Trainer  (+ researchers) | 10 |
|  | Administrations | Signing Informed consent on data collection & research | Research room | ICP and/or Trainer  (+ researchers) | 5 |
|  | Sensor donning | Bring on Wearables (Bio-sensors) | Research room | ICP and/or Trainer  (+ researchers) | 10 |
|  | Pre training Research | Registration in the MAS dashboard  Baseline questionnaire | Research room | ICP and/or Trainer  (+ researchers) | 15 |
|  | Baseline setting 1 | Biosensor measuring before VR donning  2 minutes of standing still, fixation cross on the wall | Research room | ICP and/or Trainer  (+ researchers) | 5 |
| **Training**            **95 min** | Briefing | Training pitch introduction | Training pitch | Trainer | 10 |
|  | VR donning | Bring on VR-equipment | Training pitch | Trainer | 10 |
|  | Calibration | VR-calibration | Training pitch | Trainer  (+  operator) | 5 |
|  | Tutorial | Demo scenario  Learning to use & getting used to the MR equipment  Short intro before the scenario | Training pitch | Trainer  (+ operator) | 5 |
|  | Baseli*n*e setting 2 | Heartrate variability measuring after the tutorial | Training pitch | Trainer  (+ researchers) | 5 |
|  | Trial run 1 | Country road Scenario  Short intro before the scenario  Run scenario  Data upload | Training pitch | Trainer  (+ operator) | 10 |
|  | Research run 1 | Questionnaires / interviews | Training pitch | Trainer  (+ researchers) | 10 |
|  | Hot wash run 1 | Hot wash debrief | Training pitch | Trainer | 5 |
|  | Trial run 2 | Tunnel Scenario  Short intro before the scenario  Run scenario  Data upload | Training pitch | Trainer  (+ operator) | 10 |
|  | Research run 2 | Questionnaires / interviews | Training pitch | Trainer  (+ researchers) | 10 |
|  | Hot wash run 2 | Hot wash debrief | Training pitch | Trainer | 10 |
|  | Sensor Doffing | Remove equipment & measurement tools  Disinfection VR equipment (no impact on timeline) | Training pitch | Trainer  (+ researchers) | 5 |
| **Post training**    **55 min** | AAR* | Debriefing and summary of the training  Evaluate the Stress dashboard + smart scenario control | AAR-room | Trainer | 25 |
|  | Post training research | Research questionnaires, quick interviews | AAR-room | Trainer  (+ researchers) | 20 |
|  | Focus Group | Informed consent for follow-up with selected participants | AAR-room | Trainer  (+ researchers) | 10 |
| ICP: institution contact person  AAR: After Action Review or Debriefing | | | | | |
